# Is a test better than no test when there is no treatment?

**DOI:** 10.1172/JCI173286

**Published:** 2023-07-17

**Authors:** Louise O. Downs

**Affiliations:** Exeter College, University of Oxford, Oxford, United Kingdom, and KEMRI-Wellcome Trust Research Programme, Kilifi, Kenya.

When I spent five months of my 18-year-old life living in rural Kenya, I vowed to return and do something “useful.” At that time, I hadn’t decided to become a doctor; I was going to become an ecologist focused on wildlife conservation. The trouble was that at university, I was drawn again and again towards human infection. I eventually earned a graduate medicine degree and began the long path towards an infectious diseases registrar post. I was awarded an academic clinical fellowship that allowed me to continue my passion for human infection and undertake research.

On beginning my academic post, I trawled around Oxford departments trying to decide which infection I liked most and where I should focus my research. This was hard, as I had very little insight and minimal lab experience. Eventually I went for the supervisor I liked best, as I had been told this was far more important than the actual topic, so even though hepatitis B was my least favorite virus in medical school because I didn’t understand the serology, I ended up in a hepatitis B research group with Professor Philippa Matthews.

It turned out that chronic hepatitis B (CHB) was a huge global health problem with complex issues focused in low- and middle-income countries (1) — here was my chance to return to Africa. I was awarded a Wellcome Trust Doctoral Training Fellowship at Oxford University to recruit and study a cohort of patients living with CHB through the KEMRI–Wellcome Trust Research Programme in Kilifi, Kenya, with the aim of understanding how we could better stratify people for treatment. CHB has variable clinical outcomes, from asymptomatic infection to severe liver scarring and cancer (2). International guidelines state that individuals with little or no liver disease should be monitored but not treated (3, 4).

These guidelines are complex, however, and depend on an algorithm of serum markers and liver imaging not available in most low-income countries, so treatment here is haphazard and not guideline based. CHB prevalence in Kenya is around 3%–5%, but estimates are wide and based on skewed populations (5). I aimed to undertake screening for HBV in those attending the local hospital for unrelated reasons to get a more accurate prevalence estimate and to characterize how serological markers relate to liver health.

The first project hurdle was a very long ethics approval process from the Oxford and Kenyan ethics boards. During this process, the question came up again and again of what I was going to do for those diagnosed with HBV. Could I treat them? Could I vaccinate their contacts? Could I refer them to secondary care? These were reasonable and important questions, but I hadn’t thought of this issue. In my mind a diagnosis was the most important outcome. With this, steps could be taken to reduce onward transmission and educate people about liver health, even without treatment. I hadn’t considered the stigma people would face within their community with such a diagnosis, or the need for them to feel “something was being done” even if treatment wasn’t necessarily justified or available.

Of course, my PhD budget didn’t allow for treatment provision, vaccination, or onward referral. Since these interventions were otherwise completely unaffordable to those being screened, ethics teams were unhappy about the idea of us testing at all. Was ignorance better when there was no treatment? We argued that to advocate for better clinical pathways for individuals living with CHB and to improve access to diagnostics and treatment, we first must describe the problem. Those diagnosed now were paving the way for the future, being tested for the greater good, raising awareness of the virus, and allowing the education and training of clinical staff about hepatitis B, although perhaps without any direct benefit to themselves. Was this enough to justify the test? We wrestled with these questions and discussed them with other teams and amongst ourselves. Other conditions had raised similar issues before treatment was available, and providing evidence of the need was paramount in catching the attention of any potential funder or health minister.

We eventually realized that CHB treatment was freely available locally, given that it was also a critical HIV medication. We opted to vaccinate only household contacts physically attending appointments but did not provide onward referral. We discovered that the onward referral pathway was a dead end, with no further treatment available, again indicating that advocacy for clinical HBV care was needed. We received ethics approval based on these answers, but it left me thinking about the legacy of research studies in low-income settings. What happens once the research team has left? Are participants left with stigma from a “diagnosis of interest” for which there is no cure? These complex decision-making processes will stay with me throughout my research career and will certainly make me a more well-rounded clinical academic as a result.

## Version 1 07/17/2023

Electronic publication
